# Evaluation of Whole Genome Sequencing for Outbreak Detection of *Salmonella enterica*

**DOI:** 10.1371/journal.pone.0087991

**Published:** 2014-02-04

**Authors:** Pimlapas Leekitcharoenphon, Eva M. Nielsen, Rolf S. Kaas, Ole Lund, Frank M. Aarestrup

**Affiliations:** 1 Division for Epidemiology and Microbial Genomics, National Food Institute, Technical University of Denmark, Kgs. Lyngby, Denmark; 2 Department of System Biology, Center for Biological Sequence Analysis, Technical University of Denmark, Kgs. Lyngby, Denmark; 3 Department of Microbiology and Infection Control, Statens Serum Institut, Copenhagen, Denmark; Facultad de Medicina, Uruguay

## Abstract

*Salmonella enterica* is a common cause of minor and large food borne outbreaks. To achieve successful and nearly ‘real-time’ monitoring and identification of outbreaks, reliable sub-typing is essential. Whole genome sequencing (WGS) shows great promises for using as a routine epidemiological typing tool. Here we evaluate WGS for typing of *S.* Typhimurium including different approaches for analyzing and comparing the data. A collection of 34 *S.* Typhimurium isolates was sequenced. This consisted of 18 isolates from six outbreaks and 16 epidemiologically unrelated background strains. In addition, 8 *S*. Enteritidis and 5 *S*. Derby were also sequenced and used for comparison. A number of different bioinformatics approaches were applied on the data; including pan-genome tree, k-mer tree, nucleotide difference tree and SNP tree. The outcome of each approach was evaluated in relation to the association of the isolates to specific outbreaks. The pan-genome tree clustered 65% of the *S*. Typhimurium isolates according to the pre-defined epidemiology, the k-mer tree 88%, the nucleotide difference tree 100% and the SNP tree 100% of the strains within *S*. Typhimurium. The resulting outcome of the four phylogenetic analyses were also compared to PFGE reveling that WGS typing achieved the greater performance than the traditional method. In conclusion, for *S*. Typhimurium, SNP analysis and nucleotide difference approach of WGS data seem to be the superior methods for epidemiological typing compared to other phylogenetic analytic approaches that may be used on WGS. These approaches were also superior to the more classical typing method, PFGE. Our study also indicates that WGS alone is insufficient to determine whether strains are related or un-related to outbreaks. This still requires the combination of epidemiological data and whole genome sequencing results.

## Introduction

*Salmonella* is a common cause of infectious disease in human and animals. *Salmonella* is classically divided into species *S.bongori* and *S.enterica*; the latter further divided into more than 2,500 different serotypes [1], [2]. It is, however, only a limited number of serovars that are responsible for most infections and in Europe, the most prevalent *S.enterica* serovars isolated from humans are Enteritidis and Typhimurium, responsible for over 75% of the human cases of salmonellosis [3]. *Salmonella* infections can occur as minor and major foodborne outbreaks (major outbreak - an outbreak that attracts intensive publicity). In order to elucidate the epidemiology and implement the control programs, reliable and rapid sub-typing is essential [4], [5]. Today, different typing methods are commonly used as a central part of the detection and investigation of *Salmonella* outbreaks, for instance, serotyping, phage typing, pulse-field gel electrophoresis (PFGE) and multilocus variable number of tandem repeat analysis (MLVA) [6]–[8]. PFGE has been the gold standard for epidemiological investigations of foodborne bacterial pathogens including *Salmonella* [9]. A drawback of PFGE is that it is unable to separate very closely related strains because the low rate of genetic variation does not significantly impact the electrophoretic mobility of a restriction fragment [6]. MLVA has major benefits in epidemiological surveillance of some *Salmonella* [10], but serotype specific protocols are needed for high discrimination.

During recent years the cost of whole genome sequencing (WGS) has decreased dramatically and the technology becomes increasingly available for routine use around the world [4], [11]. Moreover, the speed of sequencing is decreasing from several days or weeks to perhaps hours for a bacterial genome in the near future [12]. The combination of low cost and high speed of WGS, opens an opportunity for WGS to become very useful and practical in various bacterial infectious studies [13]–[15] including the routine use in diagnostic and public health microbiology [12], [16]. WGS has also been successfully used for elucidating the evolution of some *Salmonella* sub-types [15], [17]. Nevertheless, prior to implementing WGS in routine surveillance, it is essential to evaluate it compared to traditional method and to determine which analytic approaches that might be most useful for a given bacterial species and sub-type.

This study was conducted to evaluate WGS for outbreak typing of *S.enterica*. A collection of presumed epidemiologically related and un-related *S.enterica* strains were sequenced and analyzed using four different bioinformatics approaches. The outcome was evaluated according to the pre-defined expected epidemiological data and also compared to results obtained using the conventional typing method, PFGE.

## Methods

### Bacterial Isolates and Molecular Typing

*Salmonella* strains were derived from the Danish laboratory-based surveillance system of human gastrointestinal infections in 2000–2010. The procedures for isolation, identification, serotyping, antimicrobial susceptibility testing, PFGE and MLVA of the isolates included in this study have been described previously [9], [18]. The *S.* Typhimurium collection consisted of 18 isolates from 6 previously described outbreaks or clusters, primarily defined by MLVA [9], [10] and 16 strains that were expected to be epidemiologically un-related to the outbreaks. The outbreaks were selected to cover outbreaks that were restricted in time and location [10] as well as some epidemiologically challenging outbreaks (outbreak 1–3) that lasted several months [9]. The isolates from each outbreak/cluster were selected to include some of the known diversity within these (e.g. based on phage type, MLVA, PFGE as well as the time span of the outbreak). The 16 background strains were selected, so at least two isolates belonged to the same phage type as that of each of the 6 outbreaks. The set of *S*. Enteritidis consisted of 5 isolates from a couple of outbreaks and 3 background strains. The *S*. Derby collection comprised 3 isolates from a single outbreak and 2 background strains. Isolate information was included in Table 1.

**Table 1:** Epidemiological information for the 47 *Salmonella* genomes used in this study (source: human).

ID	Serotype	Received date	Outbreak/Background	Outbreak no.	Phage type	STTR9	STTR5	STTR6	STTR10	STTR3	MLVA pattern	Accession
0803T57157	Typhimurium	3/11/08	>1600 cases (Outbreak)	Outbreak 1	U292	2	11	13	9	212	JPX.0822.DK	ERR277220
0808S61603	Typhimurium	8/6/08	>1600 cases (Outbreak)	Outbreak 1	U292	2	11	11	9	212	JPX.0411.DK	ERR277226
0902R11254	Typhimurium	2/10/09	>1600 cases (Outbreak)	Outbreak 1	U292	2	11	13	9	212	JPX.0822.DK	ERR277229
000419417	Typhimurium	4/7/00	Background	–	U292	2	11	13	9	212	JPX.0822.DK	ERR274480
0207T641	Typhimurium	7/16/02	Background	–	U292	2	10	16	9	212	JPX.0779.DK	ERR277205
0808F31478	Typhimurium	8/27/08	>200 cases (Outbreak)	Outbreak 2	DT135	2	15	7	10	212	JPX.0855.DK	ERR277223
0903R11327	Typhimurium	3/10/09	>200 cases (Outbreak)	Outbreak 2	DT135	2	15	7	10	212	JPX.0855.DK	ERR277222
0508R6811	Typhimurium	8/24/05	Background	–	DT135	2	11	5	10	212	JPX.0273.DK	ERR277218
0811R10987	Typhimurium	11/28/08	Background	–	DT135	3	18	NA	20	311	JPX.1023.DK	ERR277224
0808R10031	Typhimurium	8/7/08	Background	–	DT135	2	11	11	9	212	JPX.0411.DK	ERR277225
0804R9234	Typhimurium	4/4/08	∼ 100 cases (Outbreak)	Outbreak 3	DT3	3	20	7	6	212	JPX.0767.DK	ERR277221
0810R10649	Typhimurium	10/2/08	∼ 100 cases (Outbreak)	Outbreak 3	DT3	3	20	7	6	212	JPX.0767.DK	ERR277227
0901M16079	Typhimurium	1/27/09	∼ 100 cases (Outbreak)	Outbreak 3	U292	3	20	7	6	212	JPX.0767.DK	ERR277228
0905W16624	Typhimurium	5/15/09	∼ 100 cases (Outbreak)	Outbreak 3	DT3	3	14	7	6	212	JPX.1118.DK	ERR277230
0110T17035	Typhimurium	10/30/01	Background	–	DT3	2	11	11	9	212	JPX.0411.DK	ERR277203
0505F37633	Typhimurium	5/13/05	Background	–	DT3	4	15	8	−2	111	JPX.0227.DK	ERR277213
0508R6701	Typhimurium	8/10/05	50 cases. Source: restaurant	Outbreak 4	DT104	3	11	18	17	311	JPX.0253.DK	ERR277214
0508R6707	Typhimurium	8/5/05	50 cases. Source: restaurant	Outbreak 4	NT	3	11	18	17	311	JPX.0253.DK	ERR277216
0508R6762	Typhimurium	8/23/05	50 cases. Source: restaurant	Outbreak 4	DT104	3	11	18	17	311	JPX.0253.DK	ERR277217
0210H31581	Typhimurium	10/24/02	Background	–	DT104	3	14	19	21	311	JPX.1563.DK	ERR277206
0510R6956	Typhimurium	10/19/05	Background	–	DT104	3	12	9	25	311	JPX.1580.DK	ERR277219
0408R5930	Typhimurium	8/26/04	Outbreak	Outbreak 5	DT12	4	4	14	7	211	JPX.0056.DK	ERR277210
0408R5960	Typhimurium	8/24/04	Outbreak	Outbreak 5	DT12	4	4	14	7	211	JPX.0056.DK	ERR277211
0409R5985	Typhimurium	9/8/04	Outbreak	Outbreak 5	DT12	4	4	14	7	211	JPX.0056.DK	ERR277212
0112F33212	Typhimurium	12/21/01	Background	–	DT12	4	13	13	8	211	JPX.0108.DK	ERR277204
0406R5753	Typhimurium	6/30/04	Background	–	DT12	4	17	12	7	211	JPX.0052.DK	ERR277207
0407M287	Typhimurium	7/5/04	Background	–	DT12	4	17	12	7	211	JPX.0052.DK	ERR277208
0407W47858	Typhimurium	7/7/04	Background	–	DT12	4	17	12	7	211	JPX.0052.DK	ERR277209
0508R6706	Typhimurium	8/3/05	Background	–	DT12	4	14	9	10	211	JPX.0167.DK	ERR277215
1004F19825	O:4,12; H:i: –	4/18/10	Outbreak	Outbreak 6	DT120	3	12	10	NA	211	JPX.0005.DK	ERR277232
1005R12913	Typhimurium	5/31/10	Outbreak	Outbreak 6	DT120	3	12	10	NA	211	JPX.0005.DK	ERR277233
1006R12965	Typhimurium	6/16/10	Outbreak	Outbreak 6	DT120	3	12	10	NA	211	JPX.0005.DK	ERR277234
0909R12120	Typhimurium	9/15/09	Background	–	DT120	3	12	9	NA	211	JPX.0007.DK	ERR277231
1007T38029	O:4,5,12; H:i: –	7/12/10	Background	–	DT120	3	14	7	NA	211	JPX.0974.DK	ERR277235
0905R11565	Enteritidis	5/18/09	Outbreak	Enteritidis 1	PT8	–	–	–	–	–	JEG.0001.DK	ERR277236
0905R11609	Enteritidis	5/26/09	Outbreak	Enteritidis 1	PT8	–	–	–	–	–	JEG.0004.DK	ERR277237
0909R12091	Enteritidis	9/4/09	Outbreak	Enteritidis 1	PT8	–	–	–	–	–	JEG.0001.DK	ERR277238
0910R12287	Enteritidis	10/23/09	Background	–	PT8	–	–	–	–	–	JEG.0073.DK	ERR248795
0909R12018	Enteritidis	9/1/09	Outbreak	Enteritidis 2	PT13a	–	–	–	–	–	JEG.0007.DK	ERR277239
0910R12234	Enteritidis	10/8/09	Outbreak	Enteritidis 2	PT13a	–	–	–	–	–	JEG.0007.DK	ERR277240
0905R11615	Enteritidis	5/29/09	Background	–	PT13a	–	–	–	–	–	JEG.0024.DK	ERR277242
0907R11860	Enteritidis	7/29/09	Background	–	PT13a	–	–	–	–	–	JEG.0021.DK	ERR277243
0807H16988	Derby	7/10/08	Outbreak	Derby outbreak	–	–	–	–	–	–	–	ERR277244
0810W40256	Derby	10/15/08	Outbreak	Derby outbreak	–	–	–	–	–	–	–	ERR277245
0903F3864	Derby	3/11/09	Outbreak	Derby outbreak	–	–	–	–	–	–	–	ERR277246
0807T13477	Derby	7/17/08	Background	–	–	–	–	–	–	–	–	ERR277247
0810F45685	Derby	10/29/08	Background	–	–	–	–	–	–	–	–	ERR277248

### Whole Genome Sequencing

The total set of forty-seven *Salmonella enterica* genomes was selected for multiplexed, paired-end sequencing on the Illumina GAIIx genome analyzer (Illumina, Inc., San Diego, CA). The procedures for DNA and library preparation including sequencing in this study have been described previously and according to Hendriksen *et al* [13]. The paired-end reads had read length at 101 bp. The genomic data have been deposited in the European Nucleotide Archive (http://www.ebi.ac.uk/ena) under accession no. ERP002633. The raw reads can be accessed online at http://www.ebi.ac.uk/ena/data/view/ERP002633. *De novo* short read assembly was performed on the set of raw reads using Velvet [19], which is a part of the pipeline available on the Center for Genomic Epidemiology (www.genomicepidemiology.org) [20], [21]. The *de novo* assembly produced contigs with average N50 = 232,749.

A number of publicly available *Salmonella* genomic data were integrated to this study making total set of analyzed data rose to 271 genomes. A set of 39 *S*. Montevideo genomes was retrieved via Bioproject 61937 with the accession numbers AESR00000000-AESY00000000, AHIA00000000 and AHHT00000000 - AHHW00000000 [17]. Nine *S*. Heidelberg genomes were downloaded using the accession number AMBU00000000, AMBV00000000, AMBW00000000, AMBX00000000, AJGW00000000, AJGX00000000, AJGY00000000, AJGZ00000000, and AJHA00000000 [22], [23]. A set of 71 *S*. Agona were received through EMBL genomic assemblies at www.ebi.ac.uk/ena (PRJEB1064-1135) [24]. A number of 105 *S*. Enteritidis genomes were retrieved via NCBI with the accession number AHUJ00000000- AHUR00000000, ALEA00000000- ALEZ00000000, ALFA00000000- ALFZ00000000, ALGA00000000-ALGZ00000000, ALHA00000000- ALHZ0000 0000 and ALIA00000000- ALID00000000 [25].

### Pan-genome Tree

Pan-genome tree was constructed from the pan-genome matrix that composed of genes and genomes (*de novo* assembled genomes from this study) as rows and columns respectively. The matrix contains profile of 0′s and 1′s represented as the absence and presence of genes across genomes. The pan-genome tree was computed on the basis of distance between pan-genome profiles using a relative Manhattan distance. The tree can be formed by hierarchical clustering by employing an average linkage, corresponding to the Unweighted Pair-Group Method with Arithmetic mean (UPGMA) algorithm. The stability of the branching was illustrated via bootstrapping. This was implemented by re-sampling genes i.e. rows of the pan-matrix, and re-clustering these data. The bootstrap value for a split is the percentage of the re-sampled trees having a similar node, i.e. with the same two sets of leaves in the branches [26], [27].

### K-mer Tree

K-mer tree, alignment-free genome phylogeny, is constructed from the contiguous sequences of k bases called k-mers [28]. K can be any positive integer. In principle, sequences with high similarity likely share k-mers [29], [30]. Based on this idea, the *de novo* assembled genomes were split into short sequences with the size of k (k-mers). If the k-mer size is tiny, the alignment specificity of k-mers will be low. If the k-mers are too large, they will be seldom aligned. K-mers were aligned against all the genomes. The number of hits or the frequency of k-mers across genomes was constructed as a matrix. The matrix consists of k-mers and genomes (rows and columns respectively) with the frequency of k-mers hits as a profile. The hierarchical clustering was performed in order to build the k-mer tree.

### Nucleotide Difference Tree (ND Tree)

We used the well-studied *S.* Typhimurium str. LT2 as a reference genome (National Center for Biotechnology Information, accession: AE006468, length of 4,857,432 bp). The reference genome was split into k-mers of length 17 and stored in a hash table. Each read with a length of at least 50 was split into 17-mers overlapping by 16. K-mers from the read and its reverse complement were mapped until an ungapped alignment with a score of at least 50 was found using a match score of 1 and a mismatch score of −3.

When all reads had been mapped, the significance of the base call at each position was evaluated by calculating the number of reads X having the most common nucleotide at that position, and the number of reads Y supporting other nucleotides. A Z-score was calculated as Z = (X−Y)/sqrt(X+Y). The value of 1.96 was used as a threshold for Z corresponding to a p-value of 0.001. It was further required that X>10*Y.

Each pair of sequences was compared and the number of nucleotide differences in positions called in all sequences was counted. We obtained similar results by using a more strict threshold of z = 3.29, but then counting nucleotide differences at all positions called by both of the strains to be compared (data not shown). A matrix with these numbers was given as input to a UPGMA algorithm implemented in the neighbor program (http://evolution.genetics.washington.edu/phylip.html) in order to construct the tree. The ND tree approach was implemented as a pipeline tool on the Center for Genomic Epidemiology (http://www.cge.cbs.dtu.dk/services/NDtree/).

### Identification of Core Genes

The set of 2,882 *Salmonella* core genes was downloaded from supplementary data of a previous publication [2]. This set of core genes (conserved genes) was estimated based on 73 publicly available *Salmonella* genomes using a previously published clustering method, which employs single-linkage clustering on top of BLASTP alignments [31], [32]. Any genes having at least 50 percent identity and 50 percent of aligned longest sequence’s length (50/50 rule) were considered as a gene cluster [31], [33]. The gene clusters that were found in all genomes were collected as a core gene.

### SNP Tree

Single nucleotide polymorphisms (SNPs) were identified using a genobox pipeline available on the Center for Genomic Epidemiology (www.genomicepidemiology.org) [34]. The pipeline consists of various freely available programs. Basically, the paired-end reads from each isolates were aligned against the reference genome, *S.* Typhimurium str. LT2, using Burrows-Wheeler Aligner (BWA) [35]. The average depth coverage was 74. SAMtools [36] ‘mpileup’ command and bedtools [37] were used to determine and filter SNPs. The qualified SNPs were selected once they met the following criteria: (1) a minimum coverage (number of reads mapped to reference positions) of 20; (2) a minimum distance of 20 bps between each SNP; (3) a minimum quality score for each SNP at 30; and (4) all indels were excluded. The qualified SNPs found within *Salmonella* core genes were ultimately used to make SNP tree because SNPs within the non-core reflect the high proportion of mobile or extra-chromosomal elements, including prophage and genomic islands [14], [38].

SNP tree was not only constructed from raw reads but also from contigs or assembled genomes. We used the software package called MUMmer version 3.23 [39]. An application named Nucmer (which is a part of MUMmer) was introduced to align each of contigs to the reference genome. SNPs were determined from the resulting alignments with another MUMmer application called “show-snps” (with options “-CIlrT”). The final set of SNPs was filtered using the following criteria; (1) a minimum distance of 20 bps between each SNP; (2) all indels were excluded.

For each genome, the final qualified SNPs for each genome were concatenated to a single alignment relatively to the position of the reference genome by an in-house perl script. If SNP is not found in the reference genome or the base coverage is less than a minimum setting (20 coverage), it is interpreted as not being a variation and the corresponding base in the reference is expected [34], [40]. Subsequently, multiple alignments were employed by MUSCLE from MEGA5 [41]. SNP tree was constructed by MEGA5 using maximum parsimony method [41]. Bootstrapping is frequently used to exhibit the reliability of the branching in a tree. From each sequence, *n* nucleotides are randomly chosen with replacements. These constitute a new set of sequences. A tree is then reconstructed and the tree topology is compared to that of the original one. This procedure of resampling the sites and the subsequent tree reconstruction is repeated 1000 times, and the percentage of times each interior branch is given is noted as bootstrap-value.

## Results

The evaluation data consisted of a set of 34 genomes and a set of 47 genomes. The former set contained 34 *S*. Typhimurium strains which 18 isolates were epidemiologically related outbreak strains from 6 different outbreaks, whereas 16 isolates were un-related strains (background or sporadic isolates). The latter set comprised 34 *S*. Typhimurium from the previous set, 8 *S*. Enteritidis of which 5 isolates were outbreak related strains from a couple of outbreaks and 3 were background strains and 5 *S*. Derby of which 3 isolates were outbreak related strains from the same outbreak and 2 isolates were background strains (Table 1).

The performance of typing methods was measured by percentage of concordance. The 100% concordance means all outbreak-related strains from a particular outbreak clustered together and separated from any background isolates.

### Traditional *Salmonella* Typing

Pulsed-field gel electrophoresis has been used as a standard procedure for epidemiological outbreak investigations of *Salmonella* [6]. Nonetheless, PFGE gave less discrimination power than WGS typing when applied to closely related strains, e.g strains with the same phage type. Some strains from different outbreaks were grouped together and some outbreak strains were mixed with background isolates (Figure S1).

### Whole-genome *Salmonella* Typing

#### Pan-genome tree

**Figure 1:**
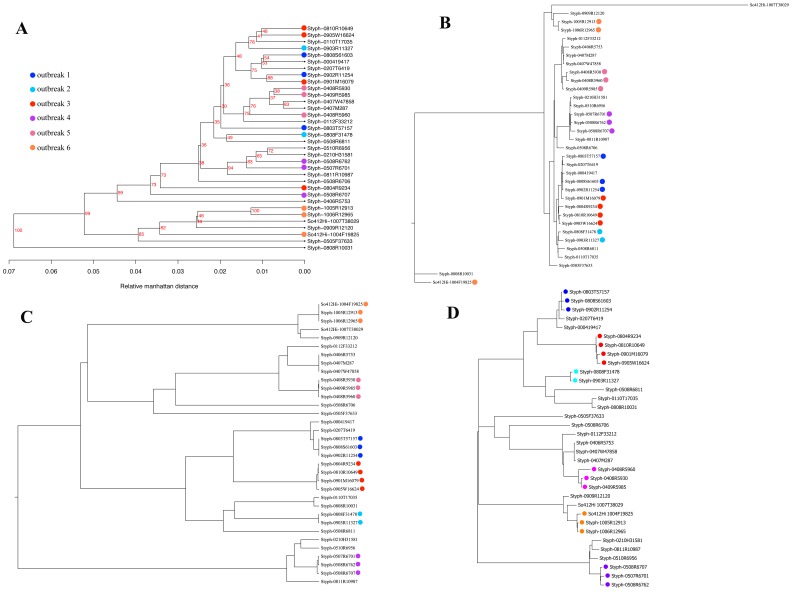
WGS typing results for the set of 34 genomes. (A) pan-genome tree, (B) K-mer tree, (C) nucleotide difference tree and (D) SNP tree. The tested set consists of outbreak-related strains displayed with color label and non-related outbreak strains shown without coloring. The outbreak strains were labeled according to the six different outbreak sources.

**Figure 2:**
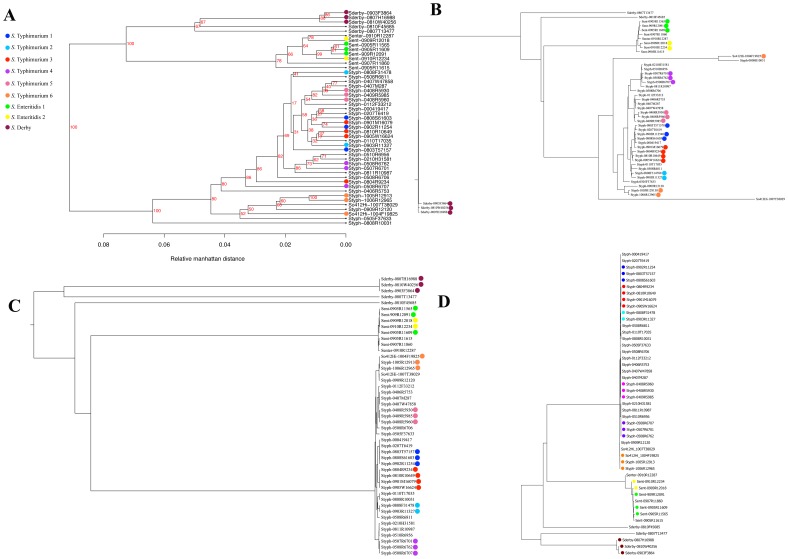
WGS typing results for the set of 47 genomes. (A) pan-genome tree, (B) K-mer tree, (C) nucleotide difference tree and (D) SNP tree. The labeled color was displayed the same as Figure 1.

The pan genome tree is the phylogenetic tree based on the profile of presence and absence of genes across genomes [2], [26], [27]. For the set of 34 genomes, the tree failed to cluster the outbreak strains into the corresponding groups of six different outbreak sources (Figure 1A). The tree only gave the reliable cluster for *S*. Derby outbreak strains (Figure 2A). Additionally, some different outbreak strains were mixed together. This method showed 65% and 64% concordance for the set of 34 and 47 genomes respectively. This is relatively low compared to the performance from other approaches (Table 2). However, the pan-genome tree revealed high performance for clustering strains according to their phage type (Figure S2).

**Table 2:** Evaluation results.

WGS typing methods	Percentage of concordance		Time (Minutes per genome)	Reference basedmethod	Type of input
	34 isolates	47 isolates			
Pan-genome tree	65	64	13	Reference free	Contigs
K-mer tree	88	89	5.2	Reference free	Contigs
Nucleotide difference tree	100	91	15	Reference-based	Raw reads
SNP tree (raw reads)	100	91	20	Reference-based	Raw reads
SNP tree (contigs)	100	89	5.5	Reference-based	Contigs

#### K-mer tree

**Figure 3:**
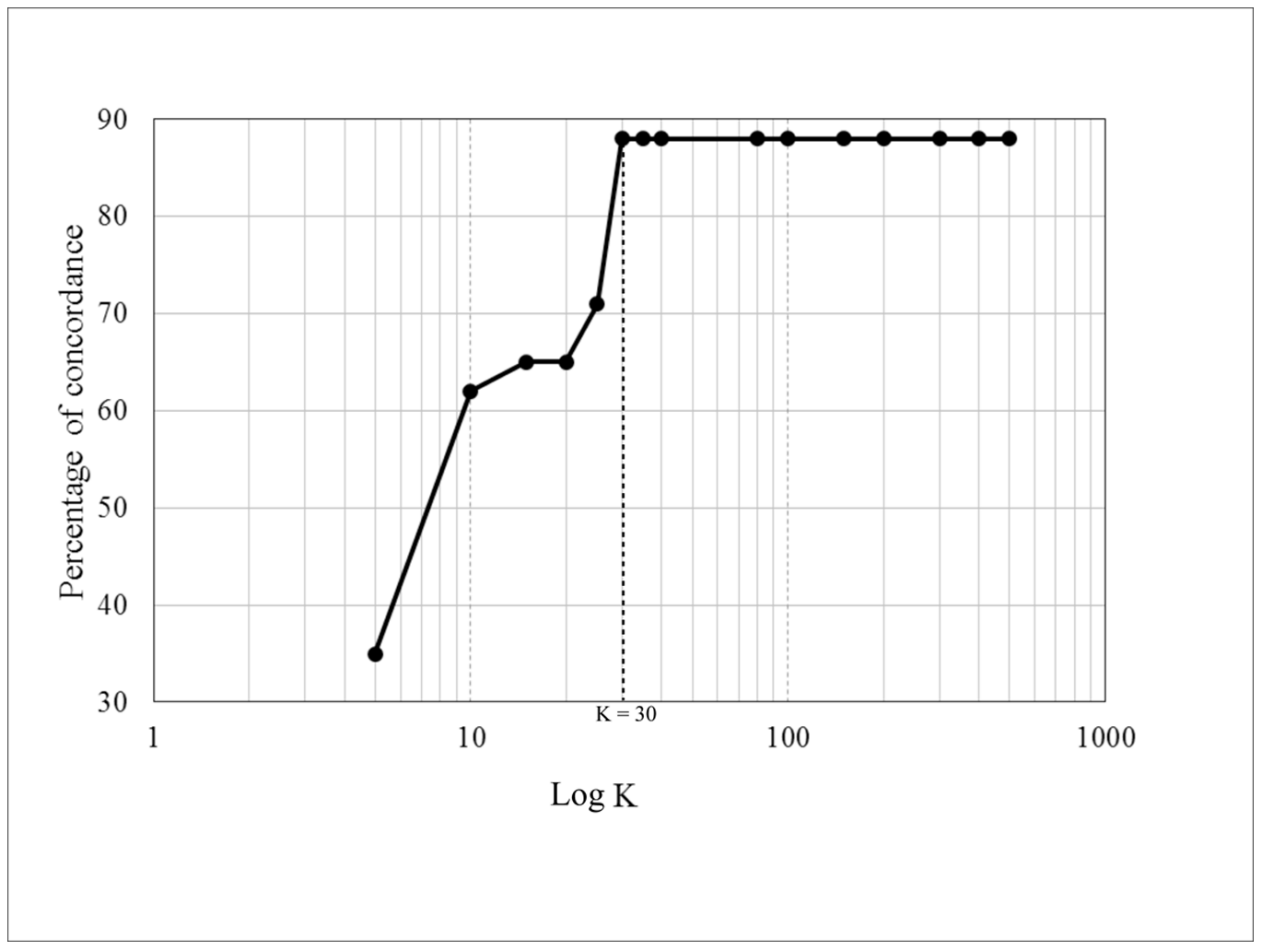
Percentage of concordance of k-mer tree on various size of k. This evaluation was conducted on the set of 34 *S*. Typhimurium.

K-mer tree was constructed from the frequency profile of k-mers across the selected genomes. The size of k is a sensitive factor for the performance of k-mer tree. A number of various k were evaluated on the set of 34 *S*. Typhimurium. Figure 3 showed an increase in the percentage of concordance with increasing k value. There was a rise in the concordance to a level of 88% concordance at k = 30. The percentage remained at this level when k>30 suggesting that this range of k achieved the highest performance of k-mer tree. Therefore, we chose k = 35 to build the final k-mer tree.

Figure 1B showed that k-mer tree gave higher resolution and more reliable tree than the pan-genome tree. However, some outbreak-related isolates were mixed up with the background strains (Figure 1B). Interestingly, the expanded tree in Figure 2B was capable to place the *S*. Enteritidis outbreak strains into two distinct clusters according to their outbreak groups. The tree also succeeded with clustering *S*. Derby outbreak strains. Nevertheless, the k-mer tree exhibited 88% and 89% concordance for the set of 34 and 47 isolates respectively (Table 2). The time consuming of k-mer tree was only 5.2 minutes per genome (including the time for assemble process). This is the fastest method compared to the others.

#### Nucleotide difference tree

As a baseline, we implemented a simple approach, the nucleotide difference tree (ND tree), which based on nucleotide difference between a pair of read mapped reference genomes. For the set of 34 *S*. Typhimurium, the ND tree classified outbreak-related strains into six obvious clusters (Figure 1C) with 100% concordance (Table 2). Thus, the typing ability of the ND tree was superior to the pan-genome tree and the k-mer tree. For the set of 47 genomes, the performance of the ND tree was slightly reduced (Figure 2C). The percentage of concordance decreased from 100 to 91% (Table 2).

#### SNP tree

SNP tree was computed from concatenated qualified SNPs identified from mapping raw reads to core genes of the reference genome [14], [38]. From figure 1D, the SNP tree clustered *S.* Typhimurium outbreak-related strains into six clusters with 100% concordance (Table 2) and furthermore differentiated them accurately from the background isolates. For the set of 47 genomes, SNP tree was able to categorized *S*. Derby isolates but unable to ultimately classify the *S*. Enteritidis strains (Figure 2D). The percentage of concordance was dropped from 100 to 91% (Table 2). This is due to the choice of reference genome, SNP tree and ND tree were able to cluster *S*. Enteritidis outbreak strains concordantly by applying publicly available *S.* Enteritis str. P125109 as a reference genome (data not shown). On average, 4.69 Mb of reference genome was covered by *S*. Typhimurium genomes meanwhile the reference genome was mapped with 4.63 Mb and 4.60 Mb when adding *S.* Enteritis and *S*. Derby.

**Figure 4:**
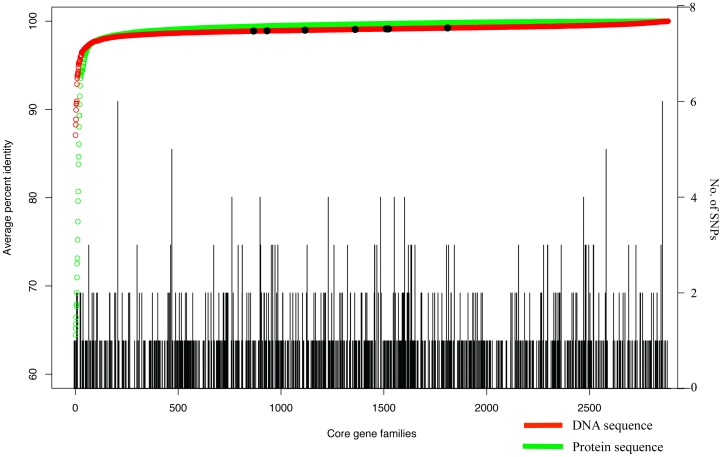
Distribution of SNPs across *Salmonella* core genes. Black bars represent number of SNPs at each core gene. Red and green small circles are core genes in the form of DNA and protein sequences respectively. The seven black dots represent house-keeping genes for MLST analysis of *Salmonella*.

The performance of SNP tree from raw reads was slightly higher than the one from contigs but constructing the SNP tree from contigs was faster (Table 2). In addition, the identified SNPs were distributed thoroughly across core genes of the reference genome (Figure 4) suggesting that the mutation occurred randomly through the core genes.

**Figure 5:**
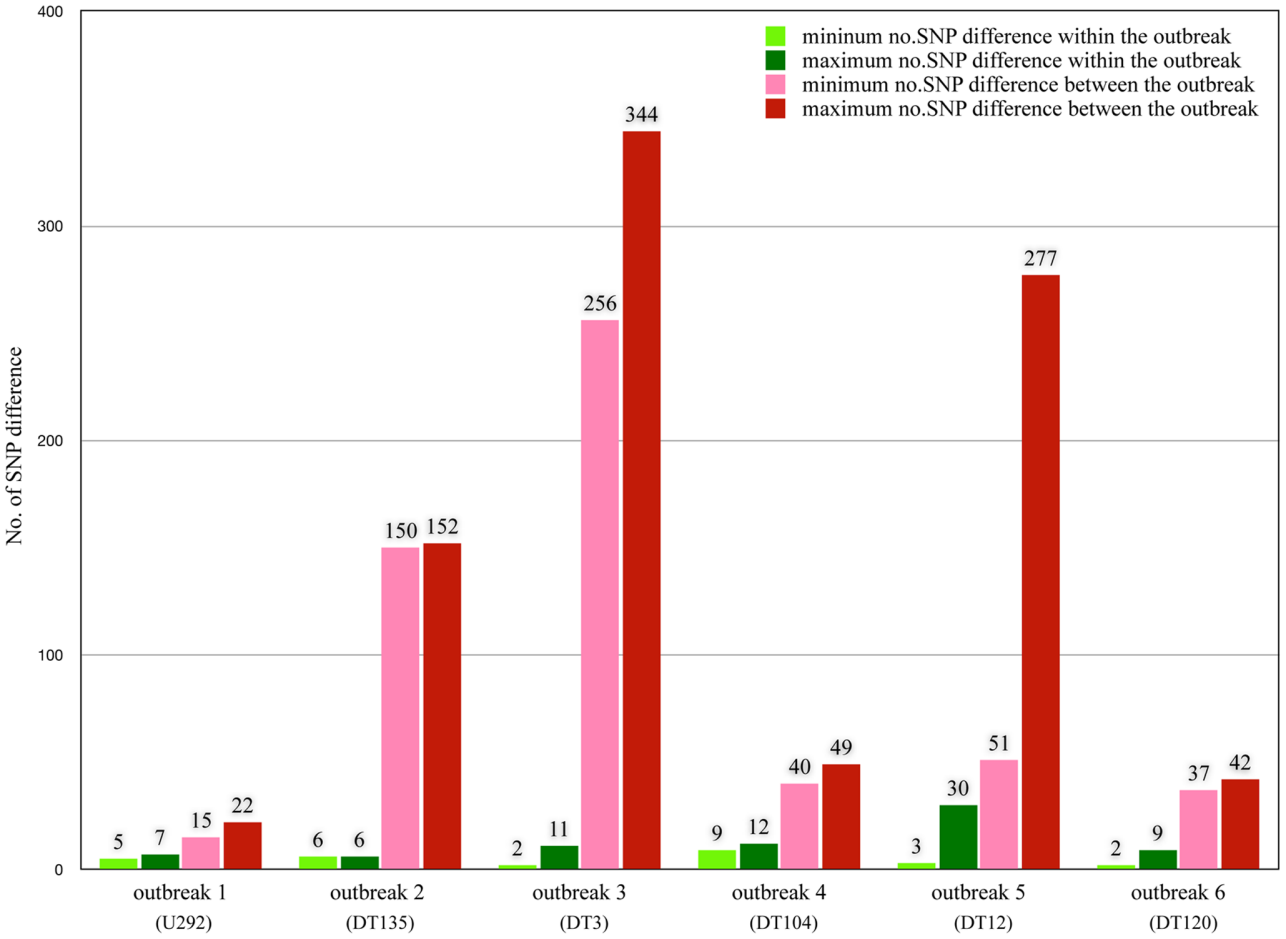
Minimum and maximum number of SNP difference. Green shaded bars show the minimum and maximum number of SNP difference between isolates within outbreaks and red shaded bars represent the number of SNP difference between outbreak-related isolates and background isolates.

Figure 5 revealed that minimum and maximum number of SNP difference within the outbreak strains were significantly less than those numbers between outbreak-related isolates and background isolates. The number of SNP difference between isolates within outbreaks ranged from 2 to 12 except the outbreak 5 (DT12) where the maximum number was relatively high (3–30 SNPs). Besides, the number of days within outbreak strains was unrelated to the number of SNP difference (Figure S3) and this relation seems to be random.

### Comparison with Published Studies

**Figure 6:**
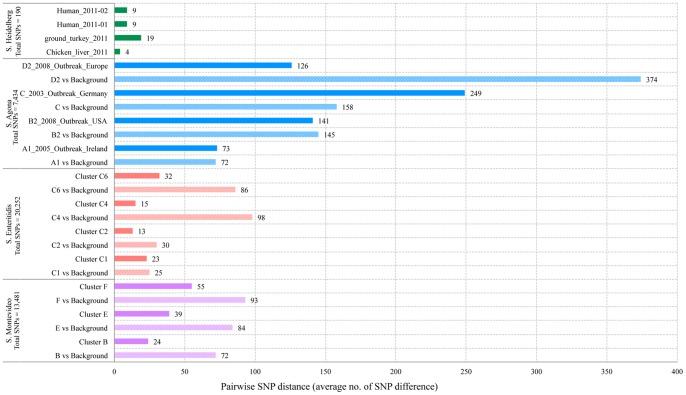
The pairwise SNPs distance. This is the average number of SNP difference between strains within outbreaks and between outbreak-related strains and background strains from the four published dataset.

Four publicly available *Salmonella* outbreak dataset were integrated and analyzed by SNP approach. These data comprised of background and outbreak-related strains except *S.* Heidelberg that contained only outbreak strains. An average number of SNP difference or pairwise SNP distance between strains within outbreaks and between outbreak-related strains and background strains were summarized in Figure 6. *S*. Montevideo and *S*. Enteritidis supported our finding that a SNP distance within outbreak strains was less than that between outbreak and background strains. Interestingly, *S.* Agona showed the higher number of SNP difference within outbreak strains and these numbers from two sub-outbreak clusters were higher than the SNP distance between background and outbreak strains. The number of SNP differences between strains within an outbreak is likely to vary for each serotype making it difficult to find the threshold for the case definition of an outbreak.

We reproduced SNP tree and k-mer tree based on 271 genomes from publicly available *Salmonella* genomes together with the genomes under study (Figure S4A and S4B). It was not possible to reproduce the tree by ND tree because most of the published data are assembled genomes and the ND tree was invented primarily for raw reads. The reproduced trees from SNP and k-mer formed distinct clusters according to serotypes. However, combining different serovar strains, k-mer and SNP trees illustrated the similar tree topology of *S*. Typhimurium cluster as they showed in Figure 1B and 1D respectively. Nonetheless, the reproduced SNP tree exhibited less resolution than the tree constructed from the strains with identical serovar as in Figure 1D.

## Discussions

The objective of this study was to determine the strengths and drawbacks of WGS using different analytic approaches compared to traditional typing method, PFGE, for retrospectively outbreak typing of *Salmonella*. A set of thirty-four human *S*. Typhimurium strains from six different outbreaks together with background strains plus eight *S*. Enteritidis isolates from two outbreaks and five *S*. Derby strains from a single outbreak were used as test sets. A number of recent studies have already used WGS for epidemiological typing of single outbreaks [13], [14], [17]. However, these studies have only used SNP analysis and not other analytic procedures. We evaluated different of analytical approaches on the WGS data set and compared to PFGE typing - the gold standard method for epidemiological studies. In our study, WGS based typing using SNP tree and ND tree was able to compete with PFGE for outbreak clustering.

The performance of the four selected WGS based typing methods was validated based on the outbreak related *Salmonella enterica* strains. Pan-genome tree failed to perform accurate clusters as the variation in protein level among the outbreak strains was not appropriate for outbreak typing, although the pan-genome tree showed meaningful clusters corresponding to phage types. This could be due to the content of prophages. The k-mer tree gave the expected clustering but was still unable to employ the complete outbreak typing. Interestingly, the k-mer tree revealed a better clustering when combining *Salmonella* strains from different serovars. This is most likely because the k-mer tree is independent from the reference genome. Another advantage of k-mer analysis is that the frequencies-based approach is much faster. Thus, it is expected to be applicable for both closely and more distantly related strains with very short time consumption for analysis. On the other hand, a deficiency is the loss of information as the huge amount of DNA sequence data is condensed into a vector of k-mer counts. Furthermore, The order of k-mers in compared sequences is neglected [30]. The nucleotide difference tree (ND tree) identified the number of nucleotide difference between a pair of raw read mapped reference genomes rather than identify the difference as SNP. This method gave the results similarly to the SNP tree. Additionally, it is important to note that SNP not being found in the reference genome is considered as not being a variation and the corresponding nucleotide from the reference is expected. This might not always be the right choice. The ND tree does not face this problem, as it does not require the concatenated sequence for alignment. ND tree was found to be somewhat sensitive to its setting. In initial calculations the mismatch score was set to −1, and in this tree all *S*. Enterititis and *S*. Derby strains became identical (data not showed). The final results used a mismatch score as −3, which is also the default in the short read alignment program, BWA.

Ultimately, SNP and ND trees were equally superior methods for clustering outbreak related isolates of *S.* Typhimurium (Figure 1C and 1D). As mentioned above, ND tree was sensitive to the parameter settings, while SNP tree failed to categorize strains with different serovars because this method depends heavily on the reference genome and this has to be closely related to the strains investigated for example the reference genome should be at least the same serovar as the strains under study. Using an inappropriate reference genome will cause exceed number of SNPs which affects the final SNP tree for instance the decreasing of the percentage concordance when adding strains with different serovars from the reference genome (Table 2, SNP tree with a set of 47 genomes). In addition, SNP tree constructed from contigs exhibited slightly less concordance than the one from the raw reads. In term of speed, the SNP tree from contigs can be achieved very fast (almost as fast as k-mer tree). It might be an alternative choice of using SNP tree for real-time typing.

We found that the numbers of SNP difference between isolates within outbreaks were very small and ranged from 2 to 12 with an exception for the outbreak 5 (DT12) where the number ranged from 3 to 30 SNP differences. Comparing to publicly available *Salmonella* genomes, the SNP distance between strains within outbreaks was possibly ranged from 4 to 249 depending on serotype suggesting that finding a general threshold to define an outbreak for all *Salmonella* might not be possible. However, these numbers may be useful as an indicator of expected SNP distance in a particular serovar or a sub-outbreak cluster within serovar. Nevertheless, by using a small number of isolates from specific outbreaks, this reduced sampling may be introduce some of other variables affecting the predictions. It may take dozens of isolates to determine the actual scope or threshold of an outbreak.

Recent studies support SNP tree as an outbreak surveillance tool such as *S.* Montevideo outbreak in United States [17], [42], *S*. Enteritidis shell egg outbreak in US in 2010 [25], *S*. Agona [24] and a 2011 multistate outbreak in the US of *S*. Heidelberg [22], [23]. Nonetheless, the SNP detection and validation need to be improved, and this method needs to be further evaluated in other bacterial pathogens to elucidate the usefulness of using SNP tree. Perhaps, for further pathogens, other approaches might be the most superior beside SNP analysis. In addition, it is especially a need to determine the importance of using different sequencing platforms, different analytic procedures and different reference strains for creating the SNP trees. Moreover, the robustness of this analytical approach for cluster detection in a routine setting has to be evaluated. The fact that the tree topology may give less resolution when new strains are added might cause some problems in the interpretation in a routine setting and over time.

In our study, we were unable to find an association between time (days) of isolation and number of SNP difference between isolates belonging to the same outbreak. This contrasts studies of methicillin-resistant *Staphylococcus aureus* (MRSA) spreading between humans in hospital community, where the time and number of SNPs are correlated [14]. This might be due to the dissimilarities in the epidemiology of these bacterial pathogens. MRSA transfers from human to human within a hospital, whereas *Salmonella* has its natural reservoir in various sources, animals and human. Thus, the transmission route of *Salmonella* to human is indirect and even though two strains are isolated with a given time interval this might not entirely reflect the number of generations that they differ. Nonetheless, this observation is in agreement with that was reported by Okoro *et al* [43]. They show that the number of days (23–486 days) between isolation of index and recurrent isolates of *S*. Typhimurium from infected patients had no obvious impact on the numbers of SNP differences accumulated, and suggest the existence of groups of isolates that comprise single clonal haplotypes with virtually no genetic change over time.

The strains included in this study were selected based on detailed epidemiological information as estimated to belong or not belonging to the same outbreak. Since the true epidemiology is not known, it cannot be excluded that strains not being part of an outbreak have been falsely included or that true outbreak strains have been falsely categorized as non-outbreak related. Based on the detailed epidemiological information available and carefully selection of isolates, we do believe that the reference material reflects the true epidemiology and that the methods SNP and ND are superior to the currently used methods for epidemiological typing such as PFGE. However, only time and routine implementation of the new WGS technologies in routine investigations will provide the value of WGS as supporting outbreak detection and control.

It is also important to note that WGS is as all other typing tools to support for decision making and should always be used in combination with epidemiological and/or clinical information. For example, the different phylogenetic trees shown in this study were not meaningful without any support from epidemiological information (the color dots in the trees). Thus, it is essential to combine epidemiological data and whole genome sequencing results.

In conclusion, this study suggests that WGS and analysis using SNP and/or nucleotide difference approaches are superior methodologies for epidemiological typing of *S*. Typhimurium isolates and might be very successfully applied for outbreak detection. For the very fast but rough result, k-mer tree might meet this requirement with constructing the tree in high speed and giving high accuracy in clade level.

## Supporting Information

Figure S1 **An UPGMA band based comparison of pulsed-field gel electrophoresis (PFGE)** ***XbaI*** **profiles.**
(PDF)

Figure S2 **Pan-genome tree with phage typing labels.**
(PDF)

Figure S3 **The relation between number of days and number of SNP difference among the outbreak strains.**
(PDF)

Figure S4A **SNP tree constructed from 271 genomes from published data and** ***Salmonella*** **genomes under this study.**
(PDF)

Figure S4B K-mer tree constructed from 271 genomes from published data and *Salmonella* genomes under this study.
(PDF)
