# Supplementary material for: Evaluation of Whole Genome Sequencing for Outbreak Detection of Salmonella enterica
Source: PLoS One. 2014 Feb 4;9(2):e87991. doi: 10.1371/journal.pone.0087991 (PMC3913712; doi:10.1371/journal.pone.0087991)
Supplement: Figure S1 — An UPGMA band based comparison of pulsed-field gel electrophoresis (PFGE) XbaI profiles. (PDF) [file pone.0087991.s001.pdf]

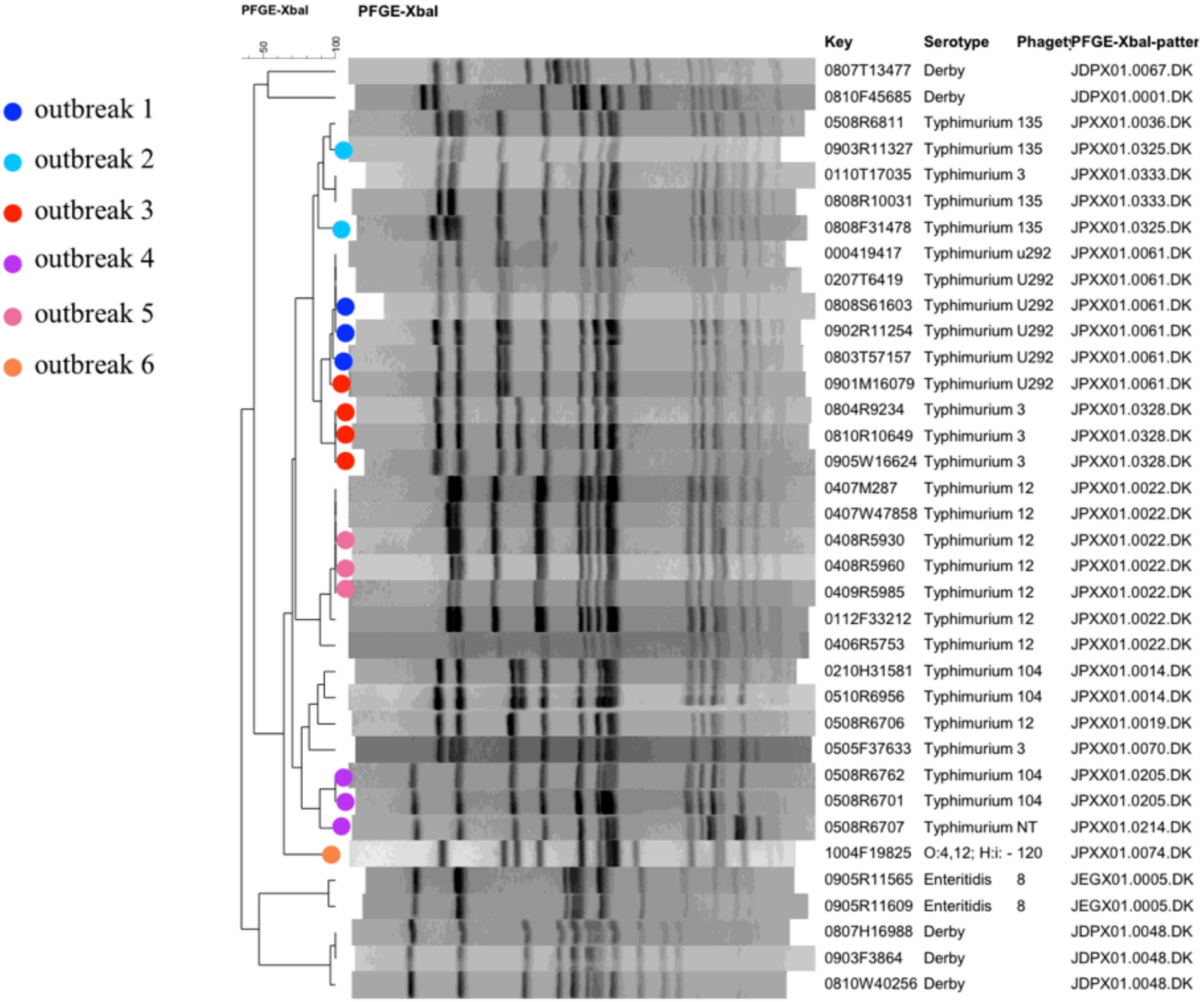

Figure S1. An UPGMA band based comparison of pulsed-field gel electrophoresis (PFGE) *XbaI* profiles.
