# Supplementary figures and images for: Evaluation of Whole Genome Sequencing for Outbreak Detection of Salmonella enterica
Source: PLoS One. 2014 Feb 4;9(2):e87991. doi: 10.1371/journal.pone.0087991 (PMC3913712; doi:10.1371/journal.pone.0087991)

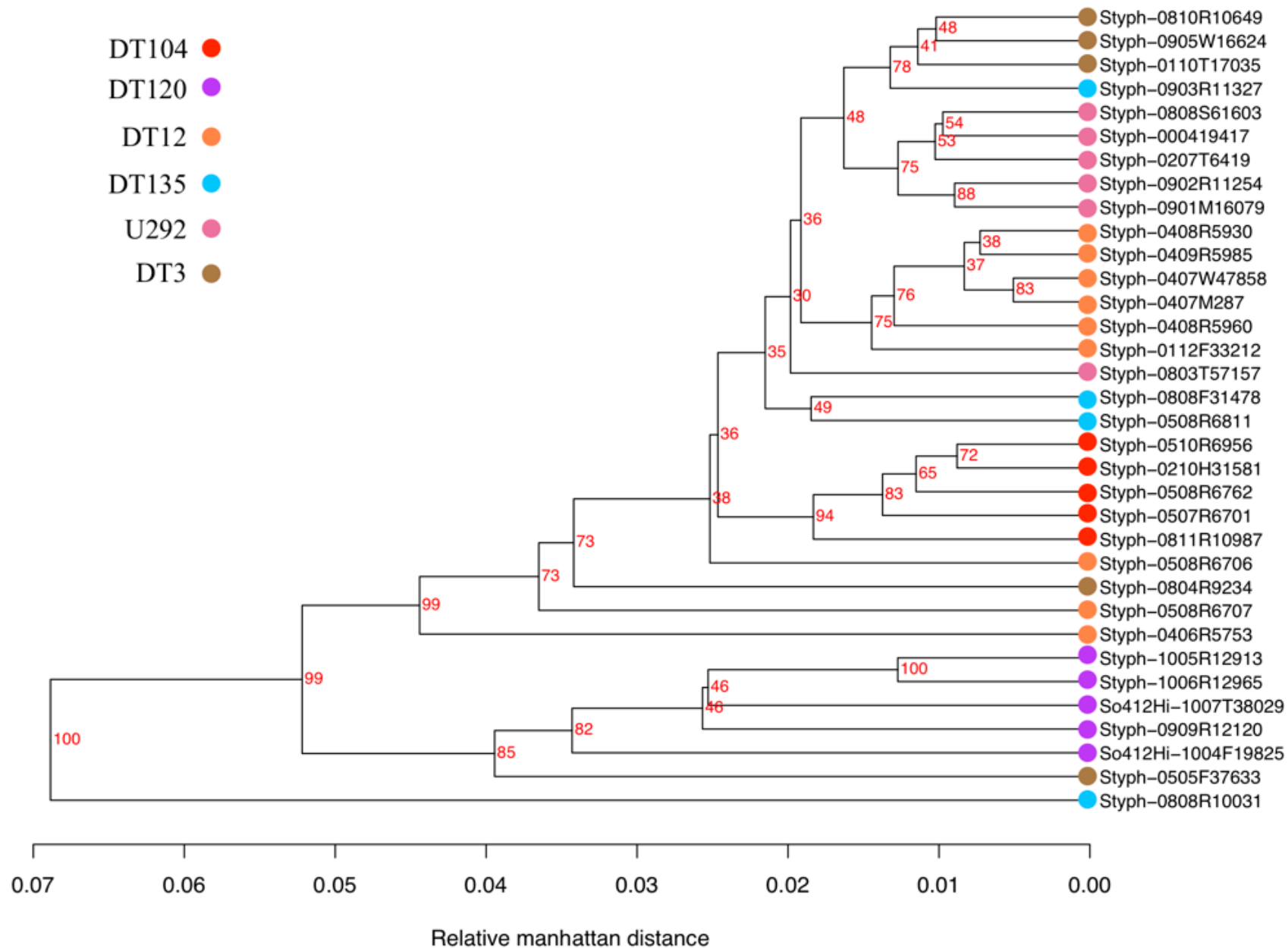

**Figure S2.** Pan-genome tree with phage typing labels.

Supplement: Figure S2 — Pan-genome tree with phage typing labels. (PDF) [file pone.0087991.s002.pdf]
