# Supplementary material for: Evaluation of Whole Genome Sequencing for Outbreak Detection of Salmonella enterica
Source: PLoS One. 2014 Feb 4;9(2):e87991. doi: 10.1371/journal.pone.0087991 (PMC3913712; doi:10.1371/journal.pone.0087991)
Supplement: Figure S3 — The relation between number of days and number of SNP difference among the outbreak strains. (PDF) [file pone.0087991.s003.pdf]

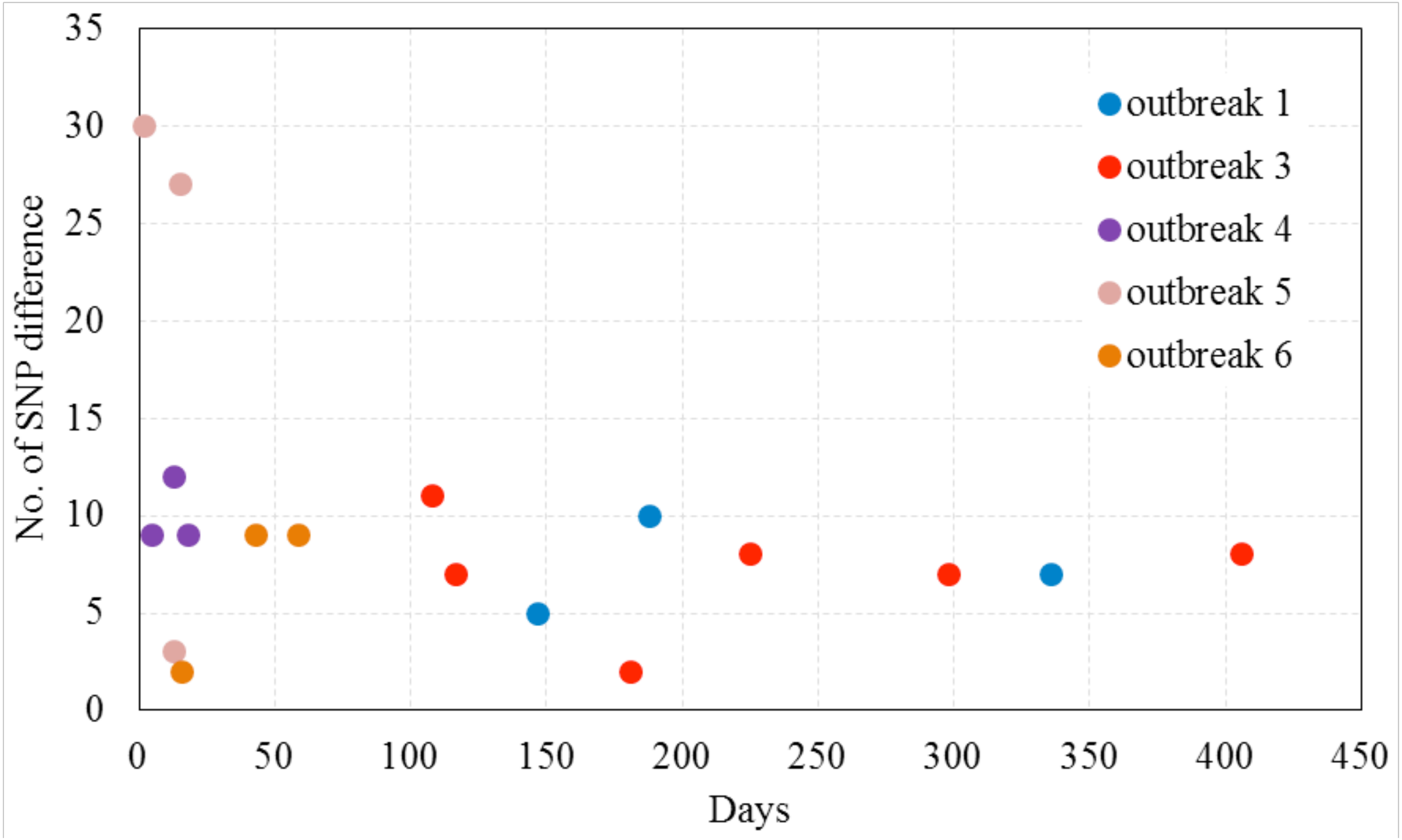

Figure S3. The relation between number of days and number of SNP difference among the outbreak strains.
