# Supplementary material for: Evaluation of Whole Genome Sequencing for Outbreak Detection of Salmonella enterica
Source: PLoS One. 2014 Feb 4;9(2):e87991. doi: 10.1371/journal.pone.0087991 (PMC3913712; doi:10.1371/journal.pone.0087991)
Supplement: Figure S4A — SNP tree constructed from 271 genomes from published data and Salmonella genomes under this study. (PDF) [file pone.0087991.s004.pdf]

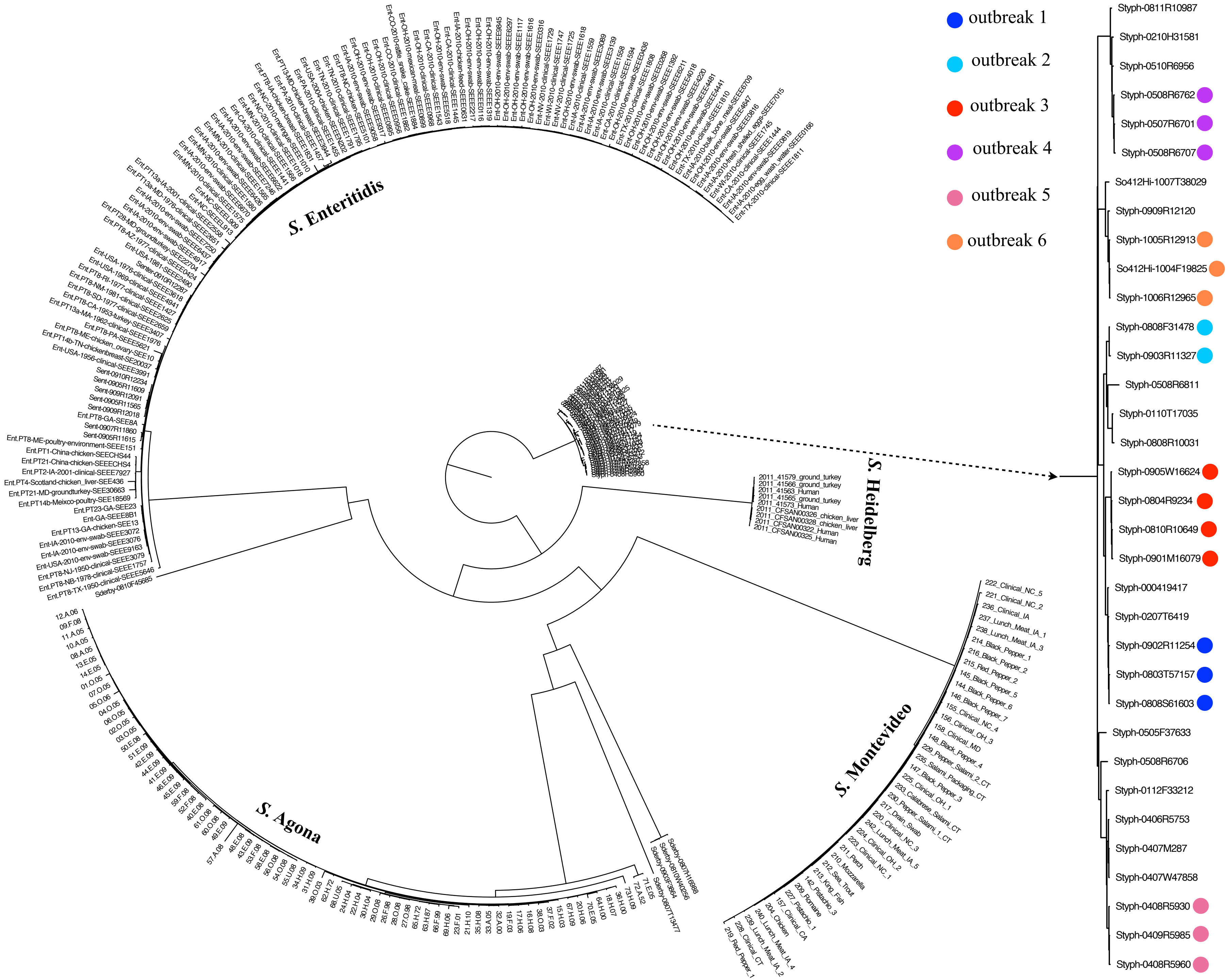

Supplementary 4A. SNP tree constructed from 271 genomes from published data and *Salmonella* genomes under this study
